# Neurodevelopmental burden in young adults undergoing opioid substitution therapy: The role of inattentive and hyperactive/impulsive symptoms

**DOI:** 10.1111/ajad.70103

**Published:** 2025-10-30

**Authors:** Maddalena Cesco, Marco Garzitto, Livia Pischiutta, Tiziana Lombardelli, Enrico Moratti, Giuliano Zamparutti, Matteo Balestrieri, Umberto Albert, Marco Colizzi

**Affiliations:** ^1^ Unit of Psychiatry and Eating Disorders, Department of Medicine (DMED) University of Udine 33100 Udine Italy; ^2^ Drug Addiction Service, Department of Mental Health and Addiction Friuli Centrale Health University Authority 33100 Udine Italy; ^3^ Department of Medicine, Surgery and Health Sciences University of Trieste Trieste 34149 Italy; ^4^ Department of Psychosis Studies, Institute of Psychiatry, Psychology and Neuroscience King's College London London SE5 8AF UK

## Abstract

**Background and Objectives:**

Attention‐deficit/hyperactivity disorder (ADHD) is recognized as a condition that can persist into adulthood, often with psychiatric comorbidities that worsen the overall prognosis. ADHD has been associated with substance use disorders (SUDs), especially through symptoms like hyperactivity and impulsivity, while the role of inattentive symptoms remains more difficult to judge. This study aimed to estimate the likelihood of ADHD in a sample of young adults with SUDs undergoing opioid agonist treatment, and to evaluate how inattentive and hyperactive/impulsive traits affect their clinical, psychological, and social functioning.

**Methods:**

57 individuals were evaluated using comprehensive clinical interviews and self‐administered questionnaires to assess characteristics of SUDs, current and childhood ADHD symptoms, levels of impulsivity, and psychiatric comorbidities.

**Results:**

Overall, 22.8% of participants were classified as possible ADHD cases (p‐ADHD). Compared to the comparison group, p‐ADHD patients exhibited more severe SUDs symptomatology and greater impairments in specific domains of adaptive functioning. They also had a higher lifetime prevalence of social anxiety disorder, unspecified Diagnostic and Statistical Manual of Mental Disorders fifth edition (DSM‐5) diagnoses, and clinically relevant impulsivity. Further, p‐ADHD individuals were more likely to experience limitations and craving, with the inattentive symptoms significantly mediating these associations.

**Discussion and Conclusions:**

ADHD frequently co‐occurs in SUDs patients in OAT and can worsen the SUDs clinical presentation. Moreover, inattentive symptoms may play a prominent role in SUDs development.

**Scientific Significance:**

This study suggests that different ADHD presentations influence SUDs manifestations, challenging the idea that impulsivity is the main contributor in SUDs.

## BACKGROUND

It is widely recognized that attention‐deficit/hyperactivity disorder (ADHD) tends to persist into adulthood, either fully manifested or with symptoms expressed at a sub‐threshold level.^1^ The continued presence of ADHD into adulthood has been found to range from 15%, when meeting the full diagnostic criteria, to 65%, when considering a partial remission of symptoms.^2^ The prevalence of ADHD diagnosis in the adults across 20 countries has been estimated at 2.8%, ranging 1.4%–3.6%.^3^

Frequently, ADHD diagnosis presents with a comorbid psychiatric condition, with a lifetime co‐morbidity rate of 60%–80%.^1^ Comorbidities have been reported to predict a poorer outcome^4^ and pose a challenge in terms of differential diagnosis, leading to underdiagnosis of ADHD and inadequate treatment of its core symptoms.^1^, ^5^ In particular, ADHD has been associated with substance use disorders (SUDs),^5^ with a recent meta‐analysis reporting a prevalence of 21%.^6^ ADHD has been found to present with high rates of use of both stimulant and sedative substances, with a prevalence of 19% in people with cocaine‐associated SUDs, 18% for opioid‐associated, and 25% for alcohol‐associated.^6^ In addition, it has been observed that 33.1% of patients with SUDs in methadone‐maintenance treatment had suffered from ADHD during childhood,^7^ making it one of the SUDs with the greatest ADHD comorbidity.

Evidence suggests that ADHD exacerbates many SUDs manifestations, such as illness duration, severity, and nonremission,^8^ also reinforcing the emerging idea that psychopathological symptoms exist on a continuum, with increasing severity linked to worsening functional and cognitive outcomes.^9^ In particular, treatment‐seeking SUDs patients with ADHD are at higher risk for additional psychiatric comorbidities than those without ADHD, especially externalized disorders.^10^ Also, SUDs patients with ADHD have been found to present with a higher intensity of craving for heroin than those without ADHD, require higher methadone dosages to inhibit withdrawal symptoms,^11^ and show a higher risk of treatment dropout.^12^

Further findings suggest a strong link between SUDs and specific ADHD presentations, particularly hyperactive/impulsive symptoms.^13^ Recent evidence indicates that impulsivity increases vulnerability to opioid use disorder and affects treatment outcomes.^14^ Also, a study conducted in a population‐based sample of twins found that hyperactivity/impulsivity in adolescence, referred to as more externalizing behavior, contributes to subsequent initiation of all types of substances, leading to either nicotine dependence and cannabis use.^15^ On the other hand, the contribution of inattentive symptoms of ADHD remains more difficult to judge, and these symptoms seem to be less frequently associated with substance use patterns.^13^ However, how the different ADHD presentations may be implicated in the progression and outcome of SUDs need further investigation.

Based on these premises, this study aimed to investigate (i) the likelihood of ADHD, based on self‐reported symptom patterns, in a sample of young adults diagnosed with severe SUDs (i.e., undergoing opioid agonist treatment, OAT), (ii) the impact of potential ADHD comorbidity on the overall clinical presentation, and (iii) whether there is a specific contribution of current inattentive or hyperactivity/impulsivity symptoms.

## METHODS

### Study design and participants

An observational study was conducted at the Drug Addiction Service of the Friuli Centrale Health University Authority, Udine, Italy. Participants were included if: (i) aged 18–30 years; (ii) in OAT with methadone or buprenorphine/naloxone; and (iii) with current clinical stability, as established by the referring physician. Exclusion criteria were the presence of: (i) intellectual disability; (ii) major psychiatric disorders (e.g., schizophrenia); and (iii) substance‐induced acute altered mental state, ongoing at assessment. The exclusion criteria were clinically evaluated by the physician responsible for the patient's care. Eligibility to participate was extended to all patients presenting to the service meeting inclusion criteria. Assessments took place in an outpatient setting, with interviews lasting about 90 min.

The participants provided written informed consent upon their admission to the service. Individuals who declined consent or discontinued participation were subsequently excluded from the data set. Data management procedures were implemented to ensure the confidentiality, integrity, and security of the collected data. This study was conducted in accordance with the Declaration of Helsinki and approved by the Institutional Review Board of the Department of Medicine (DMED) at the University of Udine (179/2023).

### Assessment

For all participants, a medical history was collected from clinical records. The assessment was carried out using self‐ and interviewer‐administered instruments. Since the Diagnostic and Statistical Manual of Mental Disorders (DSM, 5th edition) classifies ADHD as a neurodevelopmental disorder, both current (adult) and retrospective (childhood) symptoms were assessed to evaluate the prevalence of ADHD symptoms.

### SUDs characteristics

They were assessed using the Measurements in the Addictions for Triage and Evaluation (MATE, version 2.1, in Italian). MATE is a comprehensive assessment of individuals' drug and/or alcohol problems, according to the World Health Organization (WHO) classification system of both the International Classification of Diseases (ICD, 10th version) and International Classification of Functioning (ICF).^16^, ^17^ MATE assesses ten different modules and the present study focused on five of them: Substance use (S1, recent and lifetime); Substance dependence and abuse (S4, level and severity); Activities and participation—care and support (S7, social participation and environmental support); Environmental factors influencing recovery (S8); and Craving (SQ1).^17^ In this study, modules S4 and SQ1 were used as main measures of SUDs severity, while S7 and S8 to capture functional impairment.

### ADHD current symptoms

They were measured using the WHO Adult ADHD Self‐Report Screening Scale (ASRS‐18), version 1.1, in its Italian adaptation.^18^ ASRS‐18 is an 18‐item self‐administered questionnaire assessing the frequency of symptoms experienced in the past 6 months. It comprises two sections: A (6 items; a screening based on the criterion symptoms), and B (12 items; a measure of symptom severity). Besides the symptoms count (i.e., section A), the ASRS‐18 provides two factor analysis‐derived scales: Inattention (INA) and Hyperactivity/Impulsivity (H/I), both ranging 0–36. Moderate‐to‐good internal reliabilities were reported for these scales (Cronbach *α* = .76), INA (*α* = .71) and H/I (*α* = .62).

To align with DSM‐5 criteria, we also administered the WHO Adult ADHD Self‐Report Screening Scale for DSM‐5 (ASRS‐5; updating ASRS‐18).^19^, ^20^ Its score ranges 0–24 with a cut‐off of 14 for possible current disorders.^20^ The instrument, which is very short, has not been studied in terms of internal consistency. However, The Italian validation demonstrated good diagnostic accuracy (Area Under the Curve: 0.843).

Therefore, ASRS‐5 was used to screen possible ADHD, while ASRS‐18 to quantify specific ADHD presentations (i.e., INA and H/I).

### ADHD childhood symptoms

They were measured using the Wender Utah Rating Scale (WURS), a 25‐items self‐report questionnaire that retrospectively assesses childhood (6–10 years‐old) ADHD. Its scores directly correlates with the severity of childhood ADHD^21^, ^22^ and a cut‐off of 46 was suggested for clinically relevant symptoms. A good internal consistency was reported for Italian adaptation (*α* = .888), with a very high 2‐month test–retest reliability (*r* = +0.924).

### Impulsivity

It was measured using the Barratt Impulsiveness Scale (BIS‐11), a 30‐items self‐report questionnaire that assesses dispositional impulsivity traits,^23^ with proved validity.^24^ The BIS‐11 allows for the identification of an overall impulsivity score and three factors (M, Motor; A, Attentional; NP, Non‐planning). A cut‐off of 72 has been adopted to classify an individual as highly impulsive.^24^

### Psychiatric comorbidities

They were assessed using the Mini‐International Neuropsychiatric Interview, 7th edition (MINI‐7), a semi‐structured interview designed for the diagnosis of psychiatric disorders according to the DSM‐4 and ICD‐10,^25^ and recently updated to DSM‐5. It assesses the presence of 17 of the most common and significant disorders. We selected this instrument due to its sound efficiency in administration within a clinical sample and in research settings,^25^ reducing burden to the patients. The assigned diagnoses were further subjected to a comprehensive clinical evaluation. For ease of presentation, DSM‐5 disorders clinically diagnosed were classified into broader categories.

### Statistical analyses

Univariate analyses prioritized parametric tests, with nonparametric alternatives employed whenever assumptions were not met. Parallel mediation analyses were conducted specifying PROCESS Model‐4.^26^ Confidence intervals at 95% (95% CI) for indirect effects were calculated with the suggested bootstrapping procedure (with 20,000 samples). Measures were entered without centering, with (partially‐)standardized estimates reported, whether informative. Sex (coding 1 for males) and age were included as covariates. Follow‐up linear regressions were conducted as needed (e.g., for nonmediated association). Missing data were handled pairwise in univariate analyses and multiple imputation by chained equations was used in multivariable models. Statistical significance was set at *α* = .05 (two‐tailed). Analyses were conducted in R‐4.5.1 (https://www.R-project.org), also using PROCESS‐4.3.1 macros (https://processmacro.org/index.html).

## RESULTS

### Sample description

Ninety‐five individuals aged 18–30 years were considered for participation. Within this group, 38 individuals were excluded due to not meeting the inclusion criteria, refusing to participate, or dropping out during the assessment period. Ultimately, 57 patients (24 females) were recruited. General characteristics were reported in Table 1.

**Table 1:** General characteristics of the sample (57 participants).

	Mean ± SD [min, Max] and/or *N* (%)
Age	**Years: 24.3** ± **3.54 [18, 30]**
Sex	Female: 24 (42.1%) Male: 33 (57.9%)
Nationality	Italian: 52 (91.2%)
Current relationship	Single: 39 (68.4%) In a stable couple: 16 (28.1%) Separate: 2 (3.5%)
School qualification	Middle: 31 (54.4%) High: 21 (36.8%) Degree: 5 (8.8%) Years of education: 10.5 ± 2.92 [8,16]
School failures	Any school level: 49 (86%), mean number: 1.8 ± 1.25 [0, 6] At Primary school: 1 (1.8%), mean number: <0.1 ± 0.13 [0, 1] At Middle school: 23 (40.4%), mean number: 0.7 ± 0.97 [0, 4] At High school: 37 (64.9%), **mean number: 1.1** ± **1.08 [0, 4]**
Current living situation	Family of origin: 27 (47.4%) Own/New family: 17 (29.8%) Alone: 5 (8.8%) With relatives/friends: 3 (5.3%) Other living arrangement: 5 (8.8%)
Current occupation	Employed (paid job): 26 (45.6%) Unemployed: 21 (36.8%) Student: 7 (12.3%) Other situation: 3 (5.3%)
Economic status	Financially independent (stable income): 30 (52.6%) Financially independent (unstable income): 4 (7%) Financially dependent (supported by family): 20 (35.1%) Financially dependent (supported by others): 3 (5.3%)
Medical comorbidities (nonpsychiatric)	Any condition: 17 (29.8%), mean number: 0.5 ± 0.95 [0, 3] Chronic condition: 17 (29.8%) Severe condition: 8 (14%) Debilitating condition: 5 (8.8%)
Legal issues	Past: 33 (57.9%), with incarceration: 15 (26.3%) Current: 23 (40.4%)

*Note*: Bold values indicate statistically significant differences (*p* < .05) in between‐group comparisons (i.e., with vs. without probable ADHD).

Abbreviations: Max, maximum observed value; min, minimum observed value; *N*, number of observations; SD, standard deviation.

A total of 40.4% participants reported psychiatric problems in their close family members (47.4% including nonclose relatives), and 22.8% substance use problems (36.8%). Overall, 38.6% patients had received or were receiving mental health care (since the age of 15.5 ± 5.53; range: 5–24), and 31.6% had been followed by child psychiatric services (starting when 10.3 ± 3.99 years‐old; 3–15). They first accessed addiction services at a mean age of 20.7 ± 3.41 years (15–28). At assessment, all patients were undergoing OAT, that is methadone (35 patients) or buprenorphine/naloxone (22 patients). All participants reported tobacco use (start age of 13.1 ± 2.55 years, 6–23) and lifetime traumatic events were reported by 44 participants (77.2%). Clinical characteristics are detailed in Table 2.

**Table 2:** Clinical characteristics of the sample.

			Mean ± SD [min, Max] and/or *N* (%)	
		(*N* = 57)	CG (*n*_1_ = 44)	p‐ADHD (*n*_2_ = 13)
Opioid Agonist treatment	Methadone (35 patients)	Patients: Dose (mg): Age at initiation: Duration (years):	29 70.1 ± 46.07 [15, 200] 21.9 ± 2.86 [17, 27] 3.1 ± 3.14 [0.1, 11.5]	6 61.8 ± 37.79 [16, 120] 21 ± 2.25 [18, 23] 1.1 ± 0.78 [0.2, 2.4]
	Buprenorphine/Naloxone (22 patients)	Patients: Dose (mg/4 mg): Age at initiation: Duration (years):	15 8.3 ± 5.7 [1, 18] 23.1 ± 4.41 [15, 29] 2.2 ± 2.46 [0.1, 9.2]	7 10.3 ± 7.16 [2, 16] 20.2 ± 3.15 [16, 25] 0.9 ± 0.87 [0.1, 2.5]
Other treatment (current)	Prescribed drug	Any: Benzodiazepine: Antipsychotic: Antidepressant: Stabiliser: Other:	37 (84.1%) 31 (70.5%) 15 (34.1%) 10 (22.7%) 4 (9.1%) 9 (20.5%)	10 (76.9%) 7 (53.9%) 4 (30.8%) 3 (23.1%) ‐ 2 (15.4%)
	Interventions	Psychological support: Group intervention: Therapeutic community attendance:	17 (38.6%) 5 (11.6%) 4 (9.1%)	6 (46.2%) 2 (15.4%) 3 (23.1%)
**MATE**				
S1	Primary substance^(9)^	Opioids: Cocaine: Sedative: Cannabis: Alcohol:	35 (92.1%) ‐ 1 (2.6%) 1 (2.6%) 1 (2.6%)	9 (90%) 1 (10%) ‐ ‐ ‐
	Mean years of use^(2)^	Opioids: Cocaine: Stimulants: Ecstasy: Sedative: Cannabis: Gambling: Alcohol: **Nicotine:** Other:	4.2 ± 2.87 [0.5, 11] 1.8 ± 2.44 [0, 8] 1 ± 1.71 [0, 7] 0.7 ± 1.53 [0, 7] 0.7 ± 1.79 [0, 8] 6 ± 3.97 [0, 16] 0.2 ± 0.65 [0, 4] 1.8 ± 2.75 [0, 13] **10.1** ± **3.38 [4, 16]** 0.8 ± 1.63 [0, 7]	3.9 ± 1.91 [0.5, 7] 1.4 ± 2.05 [0, 7] 1.3 ± 2.4 [0, 8] 0.8 ± 2.28 [0, 8] 1 ± 2.29 [0, 8] 5 ± 3.74 [0, 12] ‐ 1.2 ± 2.1 [0, 6] **7.5** ± **3.04 [4, 13]** 1.3 ± 2.49 [0, 8]
S4	S4.1. Dependence^(2)^	**Score (0‐7):** Above threshold ( ≥ 3):	**3.6** ± **2.8 [0, 7]** 27 (62.8%)	**5.4** ± **2.64 [0, 7]** 10 (83.3%)
	S4.2. Abuse^(2)^	**Score (0‐4):** Above threshold ( ≥ 1):	**1.7** ± **1.36 [0, 4]** 29 (67.4%)	**2.6** ± **1.38 [0, 4]** 10 (83.3%)
	S4.3. Severity of dependence/abuse^(2)^	**Score (0‐9):** **Above threshold (≥8):**	**4.5** ± **3.4 [0, 9]** **12 (27.9%)**	**6.8** ± **3.27 [0, 9]** **8 (66.7%)**
S7	S7.1. Limitations ‐ Total^(4)^	**Score (0‐76):**	**11.4** ± **7.63 [0, 31]**	**22.2** ± **7.78 [7, 32]**
	S7.2. Limitations ‐ Basic^(2)^	Score (0‐32): Above threshold ( ≥ 12):	4 ± 3.68 [0, 13] 2 (4.7%)	6 ± 4.05 [0, 12] 2 (16.7%)
	S7.3. Limitations ‐ Relationships^(2)^	**Score (0‐20):**	**3.9** ± **3.12 [0, 15]**	**6.8** ± **2.82 [3, 13]**
	S7.4. Care and support^(3)^	Score (0‐32):	3.1 ± 4.78 [0, 22]	4.5 ± 4.19 [0, 12]
S8	S8.1. Positive external influences^(2)^	Score (0‐12):	4.3 ± 2.34 [0, 10]	3.1 ± 2.91 [0, 8]
	S8.2. Negative external influences^(2)^	Score (0‐20): Above threshold ( ≥ 10):	4.6 ± 4 [0, 19] 3 (7%)	6.7 ± 4.21 [1,14] 3 (25%)
	S8.3. Need for care^(4)^	**Score (0‐20):**	**5.9** ± **3.91 [0, 16]**	**10** ± **4.22 [1, 16]**
SQ1	SQ1.1. Craving^(2)^	**Score (0‐20):** Above threshold (≥12):	**3.7** ± **5.51 [0, 18]** 6 (14%)	**9.8** ± **6.09 [0, 18]** 4 (33.3%)
**MINI‐7**				
	Any disorder^(1)^	Lifetime: Current:	39 (88.6%) 22 (50%)	12 (100%) 10 (83.3%)
	Antisocial personality disorder^(1)^	Lifetime:	27 (61.4%)	11 (91.7%)
	Depressive disorder^(1)^	Lifetime: Current:	29 (65.9%) 8 (18.2%)	10 (83.3%) 4 (33.3%)
	Panic disorder^(1)^	Lifetime: Current:	18 (40.9%) 8 (18.2%)	6 (50%) 4 (33.3%)
	Generalized anxiety disorder^(1)^	Lifetime: Current:	5 (11.4%) 9 (20.5%)	4 (33.3%) 6 (50%)
	Social anxiety disorder^(1)^	**Lifetime:** Current:	**1 (2.3%)** 2 (4.6%)	**3 (25%)** 3 (25%)
	Obsessive‐compulsive disorder^(1)^	Lifetime: Current:	2 (4.6%) 1 (2.3%)	1 (8.3%) 1 (8.3%)
	Posttraumatic stress disorder^(1)^	Lifetime: Current:	8 (18.2%) 3 (6.8%)	4 (33.3%) 3 (25%)
	Anorexia nervosa^(1)^	Lifetime: Current:	1 (2.3%) ‐	‐ ‐
	Bulimia nervosa^(1)^	Lifetime: Current:	3 (6.8%) 1 (2.3%)	2 (16.7%) 2 (16.7%)
	Bipolar disorder^(1)^	Lifetime: Current:	3 (6.8%) 2 (4.6%)	1 (8.3%) 1 (8.3%)
	Psychotic disorder^(1)^	Lifetime: Current:	‐ ‐	1 (8.3%) 1 (8.3%)
	Other/Unclassified disorder^(1)^	**Lifetime:**	**1 (2.3%)**	**3 (25%)**

*Note*: Superscript numbers in parentheses indicate the number of missing values. Bold values indicate statistically significant differences (*p* < .05) in between‐group comparisons (i.e., with vs. without probable ADHD).

Abbreviations: ADHD, Attention‐Deficit/Hyperactivity Disorder; CG, Comparison Group; DSM‐5, Diagnostic and Statistical Manual of Mental Disorders, 5th edition; MATE, Measurements in the Addictions for Triage and Evaluation, Italian adaptation, version 2.1; Max, maximum observed value; min, minimum observed value; MINI‐7, Mini‐International Neuropsychiatric Interview, 7th edition (for DSM‐5); *N*/*n*, Number of observations; p‐ADHD, Possible ADHD group; S1, Primary problem (module in MATE); S4, Substance dependence and abuse (module in MATE); S7, Activities and participation—care and support (module in MATE); S8, Environmental factors influencing recovery (module in MATE); SD, standard deviation; SQ1, Craving (module in MATE).

### ADHD‐related symptoms and impulsivity

ADHD screening results and impulsivity measures are reported in Table 3. All those measures were moderately‐to‐strongly positively correlated with each other.

**Table 3:** ADHD and impulsivity measures in the sample.

			Mean ± SD [min, Max] and/or *N* (%)	
		(*N* = 57)	CG (*n*_1_ = 44)	p‐ADHD (*n*_2_ = 13)
WURS	Childhood ADHD symptoms	**Score (0–100):** **Above threshold (≥46):**	**33.1 ± 15.86 [6, 66]** **10 (22.7%)**	**66.9 ± 13.29 [46, 89]** **13 (100%)**
ASRS‐5	Current ADHD symptoms	**Score (0–24):** **Above threshold (≥14):**	**9.4 ± 3.46 [1, 16]** **4 (9.1%)**	**17.9 ± 1.73 [16, 20]** **13 (100%)**
ASRS‐18	Current ADHD symptoms	**Number (0–6):** **Above threshold (≥4):**	**2.3 ± 1.57 [0, 5]** **9 (20.5%)**	**4.6 ± 1.04 [3, 6]** **11 (84.6%)**
	Inattention scale	**Score (0–36):**	**13.9 ± 6.26 [2, 30]**	**25.7 ± 4.31 [19, 34]**
	Hyperactivity/Impulsivity scale	**Score (0–36):**	**14.2 ± 6.74 [3, 29]**	**24.9 ± 3.86 [19, 30]**
BIS‐11	Impulsivity (total scale)	**Score (30–120):** **Above threshold (≥72):**	**67.6 ± 11.11 [47, 90]** **16 (36.4%)**	**84.3 ± 12.1 [71, 106]** **12 (92.3%)**
	Attentional Impulsiveness scale	**Score (8–32):**	**17.2 ± 3.28 [12, 25]**	**22.8 ± 2.49 [17, 26]**
	Motor Impulsiveness scale	**Score (11–44):**	**23.1 ± 5.19 [12, 33]**	**29.1 ± 6.17 [21, 39]**
	Non‐Planning Impulsiveness scale	**Score (11–44):**	**27.3 ± 5.19 [16, 39]**	**32.5 ± 5.08 [27, 42]**

*Note*: Bold values indicate statistically significant differences (*p* < .05) in between‐group comparisons (i.e., with vs. without probable ADHD).

Abbreviations: ADHD, Attention‐Deficit/Hyperactivity Disorder; ASRS‐18, WHO Adult ADHD Self‐Report Screening Scale, version 1.1I, Italian 18‐items adaptation; ASRS‐5, WHO Adult ADHD Self‐Report Screening Scale for DSM‐5; BIS‐11, Barratt Impulsiveness Scale; CG, Comparison Group; DSM‐5, Diagnostic and Statistical Manual of Mental Disorders, 5th edition; Max, maximum observed value; min, minimum observed value; *N*/*n*, number of observations; p‐ADHD, Possible ADHD group; SD, standard deviation; WHO, World Health Organization; WURS, Wender Utah Rating Scale.

Based on the requirement of symptom consistency across the WURS and ASRS‐5 assessments, reflecting past and current ADHD symptomatology, 13 patients (22.8%) were classified as possible ADHD cases (p‐ADHD). The remaining 44 were designated as the comparison group (CG). p‐ADHD patients were younger than CG ones (*t*_24.3_ =+3.96, *p* < .001), with more high school failures (*U* = 154.5, *p* = .009), and more close family‐members with psychiatric problems (*U* = 196.5, *p* < .05). Clinically, p‐ADHD patients had higher scores than CG in the whole MATE S4 module: S4.1 (*U* = 152, *p* = .027), S4.2 (*U* = 158, *p* = .036), and S4.3 (*U* = 140, *p* = .015), and reported more craving (SQ1.1; *U* = 109.5, *p* = .002). Functionally, they had more limitations (S7.1; *U* = 79, *p* < .001), especially in relationships (S7.3; *U* = 109.5, *p* = .002), and a higher need of support (S8.3; *t*_16.9_ = −3.02, *p* = .008). Also, p‐ADHD patients had a higher lifetime prevalence of social anxiety disorder (OR = 13.370, *p* = .028) and unspecified DSM‐5 diagnoses (OR = 13.37, *p* = .028). Finally, p‐ADHD participants presented with higher above‐threshold impulsivity (OR = 19.987, *p* < .001) and BIS‐11 scales (all with *p* ≤ .005), than CG.

Considering ADHD types, measured with ASRS‐18 scales, age was negatively correlated with both INA (*ρ* = −0.529, *p* < .001) and H/I (*ρ* = −0.328, *p* = .013), with females scoring higher than males on H/I only (*t*_44.4_ = + 2.04, *p* = .047). INA was also negatively correlated with duration of methadone assumption (*ρ* = −0.499, *p* = .002), age at buprenorphine/naloxone initiation (*r* = −0.662, *p* < .001), and duration of group‐treatment (*r* = −0.811, *p* = .027). Also, INA scores were unevenly distributed across current occupational categories (*K*_3_ = 9.62, *p* = .022), being higher in students (*p* = .026). H/I score was negatively correlated with age at buprenorphine/naloxone initiation (*r* = −0.533, *p* = .011) and higher among patients with close relatives suffering from psychiatric problems (*t*_46.3_ = −2.28, *p* = .027) and with access to other mental health services (*t*_52.4_ = −2.78, *p* = .007). Both INA and H/I scores were moderately positively correlated with all MATE scores for S4, SQ1, and S7 modules (+0.337 < *r* < +0.581, all *p* ≤ .02), except for S7.4. Moreover, INA positively correlated with S8.2 (ρ = +0.313, *p* = .02) and H/I with S8.3 (*ρ* = +0.354, *p* = .009). Finally, considering DSM‐5 diagnoses as assessed with the MINI‐7 and confirmed through clinical evaluation, INA scores were higher among patients with a current diagnosis (*t*_54.0_ = −2.49, *p* = .016) and, for single categories, with lifetime/current generalized anxiety disorder, current social anxiety disorder, and current posttraumatic stress disorder (all with *p* < .05). H/I scored also higher among patients with a current diagnosis (*t*_53.8_ = −3.94, *p* < .001) and, for single categories, with lifetime/current generalized anxiety disorder, and unclassified disorders (all with *p* ≤ .013).

### Mediation effects of ADHD symptoms on clinical manifestation

Correcting for age and sex, p‐ADHD participants continued to score higher than CG across MATE total limitations (S7.1; *F*_3,53_ = 5.08, *p* = .004; *B* = + 7.707, *p* = .009; *R* ^2^ = 0.224), need for care (S8.3; *F*_3,53_ = 5.79, *p* = .002; *B* = + 2.748, *p* = .044; *R* ^2^ = 0.247; with a negative effect of being male: −2.505, *p* = .024), and craving (SQ1.1; *F*_3,53_ = 4.56, *p* = .006; *B* = + 5.367, *p* = .014; *R* ^2^ = 0.205). The linear model was also statistically significant for limitations in relationships (S7.3; *F*_3,53_ = 3.23, *p* = .03), even though the group effect showed only a trend towards significance (*p* = .078). Possible mediating effects of current ADHD symptoms (i.e., INA and H/I scales) were therefore tested for these selected response variables (i.e., S7.1, S8.3, and SQ1.1).

**Figure 1:**
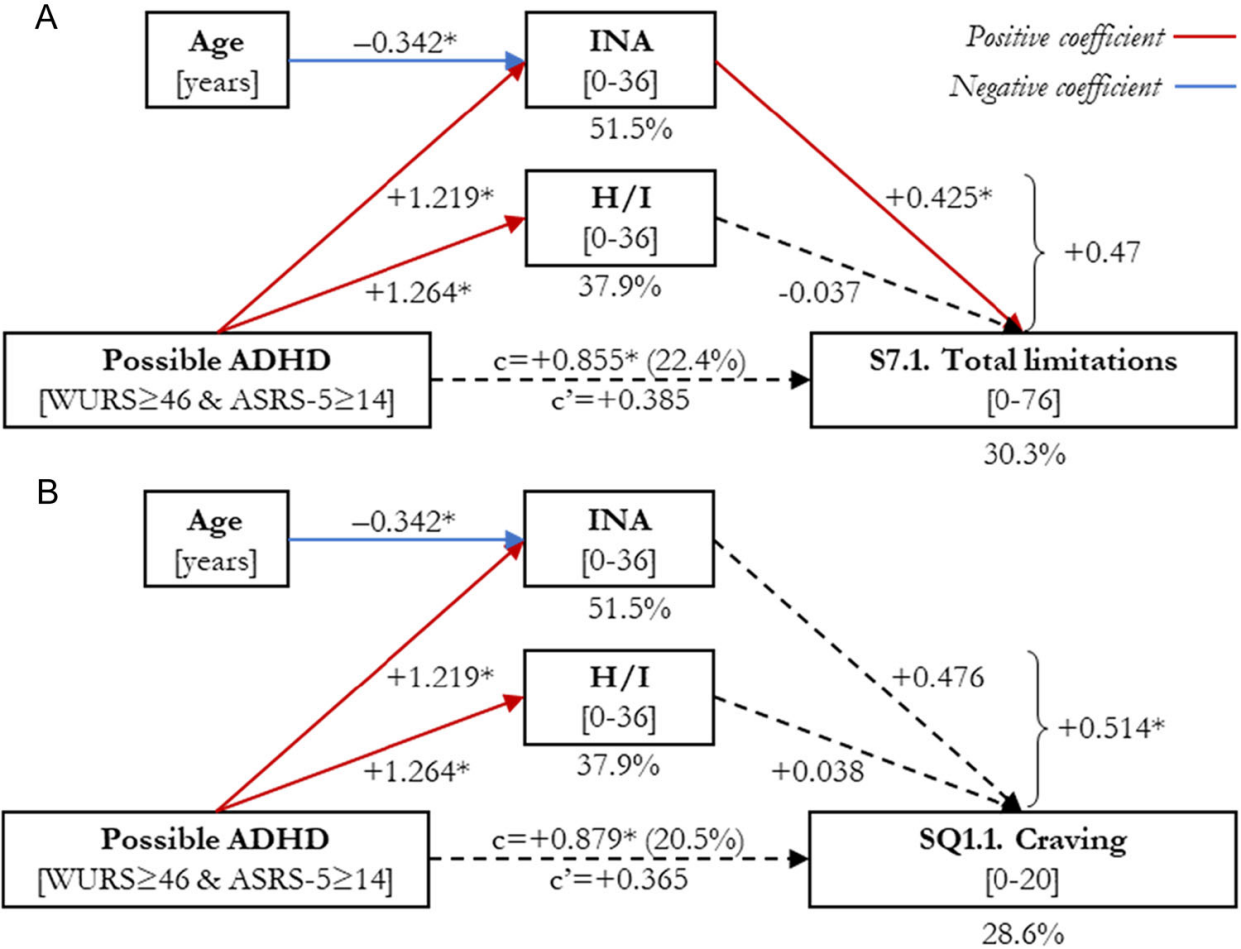
Results of mediation analyses for MATE clinical outcomes: Total limitations in activities and participation (module S7; part A) and Craving (module SQ1, part B). *Statistically significant (*p* < .05). ADHD, Attention‐Deficit/Hyperactivity Disorder; ASRS‐18, WHO Adult ADHD Self‐Report Screening Scale, version 1.1, Italian 18‐items adaptation; ASRS‐5, WHO Adult ADHD Self‐Report Screening Scale for DSM‐5; c, Partially standardised coefficient before mediation (total effect); c’, Partially standardised coefficient after mediation (direct effect); DSM‐5, Diagnostic and Statistical Manual of Mental Disorders, 5th edition; H/I, Hyperactivity/Impulsivity scale (ASRS‐18); INA, Inattention scale (ASRS‐18); MATE, Measurements in the Addictions for Triage and Evaluation, Italian adaptation, version 2.1; S7, Activities and participation—care and support (MATE module); SQ1, Craving (MATE module); WHO, World Health Organization; WURS, Wender Utah Rating Scale.

A significant mediation was observed for the total limitations score (S7.1; Figure 1a), where the effect of being a p‐ADHD patient on the response variable was partially mediated by INA (*B* = + 4.665, 95% CI: [+0.833, +9.930]), but not H/I (*B* = −0.427, [−4.784, +3.100]). When accounting for mediators, the direct effect became nonsignificant (*B* = + 3.469, [−3.439, +10.376]). Overall, INA score accounted for the 60.4% of the total effect on S7.1. Being a p‐ADHD patient had a positive effect on both INA (*B* = + 9.373, *p* < .001; *R* ^2^ = 0.515; with a negative effect of age: −0.744, *p* = .003) and H/I (*B* = + 9.695, *p* < .001; *R* ^2^ = 0.379). INA was the only statistically significant regressor in the complete model for S7.1 (*B* = + 0.498, *p* = .032; *F*_5,51_ = 4.439, *p* = .002; *R* ^2^ = 0.303).

For the need for care score (S8.3), the effect of being a p‐ADHD patient on the response variable was not significantly mediated by INA (*B* = −0.356, [−2.22, +1.618]) nor by H/I (*B* = + 0.731, [−1.091, +2.771]). Being male was the only statistically significant regressor in the complete model for S8.3 (*B* = −2.071, *p* = .044; *F*_5,51_ = 3.503, *p* = .009; *R* ^2^ = 0.256).

Finally, for the craving score (SQ1.1; Figure 1b), the direct effect of being in p‐ADHD was not statistically significant after introducing the mediators (*B* = + 2.229, [−2.509, +6.967]), whereas the indirect effect was significant (*B* = + 3.138, [+0.452, +6.263]). Although neither the effect of INA (*B* = + 2.905, [−0.075, +6.953) nor that of H/I (*B* = + 0.233, [−3.034, +2.876]) was statistically significant, the INA score was close to significance and accounted for a considerably larger portion of the total effect (54.1% vs. 4.3%). None of the regressors had a statistically significant effect in the complete model, but INA showed a trend towards significance (*B* = + 0.310, *p* = .051; *F*_5,51_ = 4.085, *p* = .003; *R* ^2^ = 0.286).

## DISCUSSION

This study aimed at estimating the frequency of ADHD traits in a sample of young adults undergoing OAT, and their impact in terms of SUDs severity and associated functioning. Following screening recommendations, 29.8% of the sample scored above the threshold on the ASRS‐5, 35.1% on the ASRS‐18, and 40.4% on the WURS. When combining ASRS‐5 and WURS, 22.8% tested positive for possible ADHD (p‐ADHD), in line with evidence from a recent meta‐analysis estimating the prevalence of ADHD in individuals with SUDs at 21%.^6^

Our findings indicate that ADHD symptoms significantly worsen SUDs presentation. In fact, p‐ADHD participants were on average 3.6 years younger than the CG, possibly reflecting and earlier SUDs onset, and were more likely to experience craving (SQ1), aligning with previous research,^11^ and struggle in controlling substance use (S4), potentially indicating a more severe clinical course.^12^ The effect of SUDs was also worse in terms of disability, with p‐ADHD individuals exhibiting greater limitations (S7.1), especially in their ability to actively engage in societal participation (S7.3), and decreased overall autonomy (S8.3). Of note, p‐ADHD patients more often reported academic failures and having family members with psychiatric problems, suggesting a more complex lifetime trajectory and a greater heredo‐familial burden. Furthermore, these findings align with evidence indicating that psychopathological manifestations, beyond the classical nosological classifications, are better conceptualized along a continuum of severity, whereby greater burden is associated with progressive deterioration in functional capacity and cognitive performance.^9^

Of interest, ADHD‐related nuclear symptoms, namely Inattention (INA) and Hyperactivity‐Impulsivity (H/I), were found to be differentially associated with specific SUDs dimensions. INA correlated with Negative External Influences (S8.2), while H/I with Care Needs (S8.3), indicating that ADHD symptomatic profiles may differentially contribute to specific SUDs vulnerabilities, possibly due to the impact of inattention on social integration and planning on one hand, and hyperactivity/impulsivity on disruptive behaviors on the other.^1^

Further, lifetime social anxiety and other/unclassified disorders were found to be highly prevalent among p‐ADHD patients (25.0% vs. 2.3%). Also, higher scores in both INA and H/I were found to be associated with having a comorbid psychiatric disorder and a lifetime and/or a current generalized anxiety disorder. Again, a differential contribution of ADHD symptomatic profiles emerged, with INA being specifically associated with current social anxiety and posttraumatic stress disorders, and H/I with other/unclassified disorders. These findings suggest a greater psychopathological burden when SUDs patients undergoing OAT with comorbid ADHD, possibly due to the impact of inattentive symptoms on anxiety spectrum disorders, reinforcing observations that anxiety symptoms, together with mood symptomatology, may hide undiagnosed or untreated ADHD.^5^

In line with evidence that impulsivity is common among both ADHD and SUDs,^27^ results indicate that dispositional impulsivity measured by BIS‐11 is more pronounced in patients with a potential dual diagnosis. In fact, stable impulsivity traits were largely prevalent among the p‐ADHD group (92.3% vs 36.4%), also considering impulsivity‐related subdomains. In contrasts, a previous study reported low impulsivity in individuals with heroin dependence, a pattern that the authors attributed as contributing to their capacity to maintain long‐term abstinence even in the absence of OAT.^28^ Conversely, all patients in our sample were undergoing treatment, therefore high impulsivity could be expected.

Finally, this study disentangled the respective contributions of INA and H/I traits, finding that inattention traits have a significantly greater effect in predicting SUDs severity. In fact, INA scores were found to partially mediate patients' global limitations and craving, accounting for a significant proportion of the variance of these clinical manifestations. Thus, these observations corroborate and extend previous evidence that ADHD symptomatology predicts a more severe SUDs clinical course, accompanied by pronounced social and psychiatric impairments,^29^ also suggesting that inattentive symptoms may play a predominant role in this relationship.

The role played by inattentive symptoms of ADHD in SUDs remains poorly investigated. In the literature, the presence of inattentive symptoms has been linked to alterations in the functioning of specific neural circuits, particularly the Default Mode Network (DMN),^30^ which is predominantly active during rest, and is thought to support self‐referential and internally oriented processes.^31^ Evidence indicates that adults with ADHD exhibit reduced sustained suppression of the DMN during value‐based decision‐making.^30^ Alterations in this network can lead to intrusions during active tasks, resulting in attention fluctuations interfering with goal‐directed processing, which has been hypothesized to be an underlying cause of ADHD attentional variability.^32^ Considering this, inattentive symptoms may be linked to increased focus on internal states and impaired goal‐directed behavior. It is therefore reasonable to hypothesize that they can lead to higher craving and challenges in maintaining abstinence and self‐control, leading to poorly planned actions.

From a different standpoint, the Research Domain Criteria (RDoC) framework adopts a transdiagnostic and neurobiological approach that conceptualizes mental illness as the result of dysfunctions in specific brain circuits rather than discrete diagnostic categories.^33^ From this perspective, it has been shown that neural systems that support adaptive and flexible cognition exhibit common vulnerability across various forms of psychopathology, suggesting shared underlying substrates.^34^ The preliminary results of our research indicate that different ADHD dimensions exert distinct impacts, suggesting separate underlying neurobiological pathways. This latter hypothesis challenges conventional psychopathological classifications and suggests the presence of shared mechanisms between different diagnoses (e.g., ADHD and SUDs) rather than independent disorders.

Eventually, evidence suggests that in adulthood ADHD is predominantly characterized by inattentive symptoms, together with inner restlessness, disorganization, and deficits in executive functioning,^35^ that may lead to greater social impairment. The present study corroborates and further extends this observation, underscoring the need for continued clinical attention and targeted interventions in adult populations.

This study benefited from several strengths, including the recruitment of a clinically well‐defined sample and the use of validated psychometric tools alongside clinical assessment. These features improve reproducibility and translational value, making them immediately informative at a clinical level.

However, several limitations should be acknowledged, such as the small sample size, the absence of diagnostic interviews for ADHD assessment, and the lack of a control group. As noted above, all patients who met the inclusion criteria were invited to participate. However, given the low‐threshold setting of the service, random sampling was not feasible, and the study therefore relied on a convenience sample. In addition, the findings concern OAT patients who, though representative of the service population, constitute a specific clinical subset, limiting the generalizability of the results to other populations. Given these constraints, the results should be regarded as preliminary and exploratory, and interpreted with appropriate caution. Accordingly, future investigations should replicate these findings in larger multisite cohorts with formal ADHD diagnosis, and clarify the differential impact of ADHD on clinical manifestations, with particular emphasis on the inattentive presentation and the underlying neurobiological mechanisms.

## CONCLUSIONS

The current study indicates that ADHD may co‐occur in SUDs patients undergoing OAT, highlighting an underrecognized dimension of comorbidity that warrants further investigation. Also, ADHD symptoms can significantly worsen the SUDs clinical presentation, suggesting the need for tailored interventions that address the heightened craving, social impairments, and susceptibility to environmental stressors. Finally, evidence indicates that inattentive symptoms may play a more prominent role than impulsive ones in shaping the SUDs clinical trajectory, in adult individuals, challenging the traditional view that impulsivity predominantly drives substance misuse.^13^, ^27^

## CONFLICT OF INTEREST STATEMENT

M.Co. has been a consultant/advisor to GW Pharma Limited, GW Pharma Italy SRL, and F. Hoffmann‐La Roche Limited, outside of this work. All other authors declare that they have no competing interests.

## Data Availability

The datasets used and/or analyzed during the current study are available from the corresponding author on reasonable request.
